# GERIATRICS MICU CO-MANAGEMENT: INTEGRATING GERIATRIC ASSESSMENTS FOR CRITICALLY ILL PATIENTS

**DOI:** 10.1093/geroni/igz038.1681

**Published:** 2019-11-08

**Authors:** Gowrishankar Gnanasekaran, Eduardo Mireles-Cabodevila

**Affiliations:** Cleveland Clinic Foundation, Cleveland, Ohio, United States

## Abstract

Programs like orthogeriatrics, geriatric cardiology have shown to improve outcomes in hospitalized geriatric patients. Our Geriatrics MICU Co-management program is a quality improvement initiative that instigates a partnership approach with critical care medicine in integrating geriatric assessments and build foundation for interdisciplinary care of critically ill patients. MICU (Medical Intensive Care Unit) protocols do not have standard geriatrics assessments integrated in clinical care. An electronic dash-board identifies high risk elderly (HRE) patients admitted at a MICU in a large teaching hospital in Northeast Ohio based on nursing specific screening triggers. A geriatrics co-management team engages in a comprehensive geriatric assessments and care transition. 386 patient were identified using HRE screening triggers in a period of 100 days. 33 % (n=131) were generated as consults for co-management. A pilot review on 131 HRE patients was conducted. 70% (n=93) patients had incident frailty. 93% (n=87) of patients with frailty were diagnosed with incident delirium. 56% (n=74) of patients were newly diagnosed with cognitive impairment. 56 % (N=74) of patients had a medication reduction. An average of 1.23 medication was changed. 85% (n =112) of patients had a warm hand off to the next level of provider on discharge. 90% (n=119) of patients notified improved self-management skills and better understanding of discharge process. The Geri-MICU program demonstrates a patient -centered approach in integrating geriatric assessments for critically ill patients and build foundation of a geriatrics-critical care task force. The program would be a mile stone in optimizing elderly care in critical care units.

